# Including frameworks of public health ethics in computational modelling of infectious disease interventions

**DOI:** 10.1098/rsfs.2025.0004

**Published:** 2025-09-26

**Authors:** Alexander E. Zarebski, Nefel Tellioglu, Jessica E. Stockdale, Julie A. Spencer, Wasiur R. KhudaBukhsh, Joel C. Miller, Cameron Zachreson

**Affiliations:** ^1^ University of Melbourne School of Mathematics and Statistics, Parkville, Victoria, Australia; ^2^ University of Oxford Pandemic Sciences Institute, Oxford, UK; ^3^ MRC Biostatistics Unit, Cambridge, UK; ^4^ Department of Infectious Diseases, The Peter Doherty Institute for Infection and Immunity, Melbourne, Victoria, Australia; ^5^ Department of Mathematics, Simon Fraser University, Burnaby, Canada; ^6^ Information Systems and Modeling Group, Los Alamos National Laboratory, Los Alamos, NM, USA; ^7^ School of Mathematical Sciences, University of Nottingham, Nottingham, UK; ^8^ Department of Mathematical and Physical Sciences, La Trobe University, Melbourne, Australia; ^9^ Australian Centre for AI in Medical Innovation, Melbourne, Victoria, Australia; ^10^ The University of Melbourne School of Computing and Information Systems, Parkville, Victoria, Australia

**Keywords:** public health ethics, ethical framework, infectious disease, computational model, intervention, vaccination

## Abstract

Decisions on public health interventions to control infectious diseases are often informed by computational models. Interpreting the predicted outcomes of a public health decision requires not only high-quality modelling but also an ethical framework for assessing the benefits and harms associated with different options. The design and specification of ethical frameworks matured independently of computational modelling, so many values recognized as important for ethical decision-making are missing from computational models. We demonstrate a proof-of-concept approach to incorporate multiple public health values into the evaluation of a simple computational model for vaccination against a pathogen such as SARS-CoV-2. By examining a bounded space of alternative prioritizations of three values relevant to public health ethics (aggregate clinical burden, equity in clinical burden, equity in adverse effects from vaccination), we identify value trade-offs, where the outcomes of optimal strategies differ depending on the ethical framework. This work demonstrates an approach to incorporating diverse values into decision criteria used to evaluate outcomes of models of infectious disease interventions.

## 1. Introduction

Frameworks of public health ethics offer guidelines for balancing competing values when making decisions that affect the lives and livelihoods of populations [1,2]. In responding to infectious disease outbreaks and pandemics, public health interventions have often prioritized preventing clinical burden from overwhelming health services. To inform decisions about interventions, public health institutions typically project how potential interventions will influence aggregate outcomes, such as the number of concurrent hospital admissions at the peak of an outbreak, the number of lives saved or the number of quality-adjusted life years (QALYs) saved. While aggregate measures, such as hospital admissions or QALYs, are insufficient for a fully utilitarian assessment of well-being, there is a clear connection with utilitarian ethical frameworks that consider aggregate outcomes, without regard for their distribution [3,4].

Evaluating public health decisions in terms of aggregate benefit has the potential to create or exacerbate inequitable distributions of benefits and burdens [5–7]. To address this issue in utilitarian public health policy, there have been efforts to design ethical frameworks and analytical techniques that can balance aggregate benefits and equitable outcomes [2,8–13]. For example, techniques such as prioritarian social welfare analysis or distributional cost-effectiveness analysis have begun to formally address deficiencies of utilitarian frameworks in terms of distributive justice [13–15].

During the COVID-19 pandemic, computational and mathematical models were widely applied to inform public health interventions. While modelling provided invaluable guidance to policymakers, it typically did not account for impacts other than aggregate clinical burden, ignoring other values relevant to public health ethics, such as equity [16]. Decision makers had to make judgements on these impacts without quantitative guidance. To help address this deficiency, modellers need to translate qualitative features of ethical frameworks into quantitative model features.

Here, we demonstrate a way to include elements of ethical frameworks within a quantitative model of vaccination against a pandemic pathogen. We consider a simple model of pathogen transmission to illustrate the representation of ethical values in an optimization problem to select a vaccination strategy. To highlight the relevance of this approach to the development of ethical frameworks that include both aggregate well-being and distributive justice, we choose to examine outcome equity and aggregate benefit. To connect our approach to a realistic case study, we implement a model of COVID-19 transmission using parameters from the literature and use expected hospitalization rates to evaluate intervention outcomes.

In §2.1, we describe our model of pathogen transmission with a variable level of vaccination in an age-stratified population. In §2.2, we cast the selection of a vaccination strategy as an optimization problem, selecting a level of vaccination that minimizes the value of a loss function which provides a quantitative description of how ‘good’ the outcome of a given intervention is. While the loss function we describe is specific to this model and choice of ethical values, the approach may be generalized. In §2.3, we describe the parametrization of our model that we use to illustrate this approach for three scenarios: one intended to reflect the transmission properties of COVID-19, and two additional scenarios with greater and smaller pathogen transmissibility in an urban population (Melbourne, Australia).

Our results are presented in §3. We first analyse the optimal level of vaccination for the COVID-19 scenario in each age group under three (extreme) ethical frameworks and then extend our analysis to a broader range of prioritization schemes for ethical values.

We then repeat this analysis for scenarios with greater and smaller disease transmission rates. For each scenario, we characterize the properties of optimal interventions over the space of ethical frameworks we consider. We then describe the qualitative and quantitative features of our results, including the presence or absence of trade-offs between values. We conclude with a discussion of the key findings and the limitations of our study.

## 2. Methods

### 2.1. Transmission model

To simulate pathogen transmission and mitigation, we use a deterministic susceptible-infected-recovered (SIR) model with imperfect vaccination (using the ‘all-or-none’ mode of vaccine failure). We consider a heterogeneous population consisting of N people divided into two groups of size $N_{1}$ and $N_{2}$. Contact occurs within and between groups (at potentially different rates). Each group can be further divided into those who are susceptible to infection, currently infectious or removed from the infectious class (and hence immune to subsequent infection).

Let $S_{i}(t)$, $I_{i}(t)$ and $R_{i}(t)$ denote the number of people in group i who are susceptible, infectious and immune at time t. Initially, there is a single infected individual (index case) in each group. Members of $S_{j}$ become infected and move to $I_{j}$ at a rate $\sum_{i}\beta_{ij}I_{i}/N_{j}$. The ith summand corresponds to an infected individual of group i infecting an individual of group j.

Infection occurs proportional to $\beta_{ij}=\beta c_{ij}$, where β is a global transmission scalar and $c_{ij}$ is proportional to the rate of contact an individual in group i has with the people in group j. Transmission may occur if the person in group j is susceptible, which has probability $S_{j}/N_{j}$. Members of $I_{i}$ recover from infection (and become immune to subsequent infection) at rate γ. Although we include only two groups (i,j∈{1,2}), the model can be readily extended to incorporate additional heterogeneity.

#### 2.1.1. Imperfect vaccination

We assume an ‘all-or-none’ mode of vaccine failure in which a proportion of people receiving the vaccine gain full immunity and the remainder are not protected against infection. For simplicity, we assume all vaccinations occur before the initiation of the outbreak, with proportions $p_{1}$ and $p_{2}$ of individuals in groups 1 and 2 being vaccinated. Each choice of how many people to vaccinate in each group, $p_{i}$, is a *vaccination strategy*. The selection of a vaccination strategy is the focus of our optimization framework, as described below.

**Figure 1.**
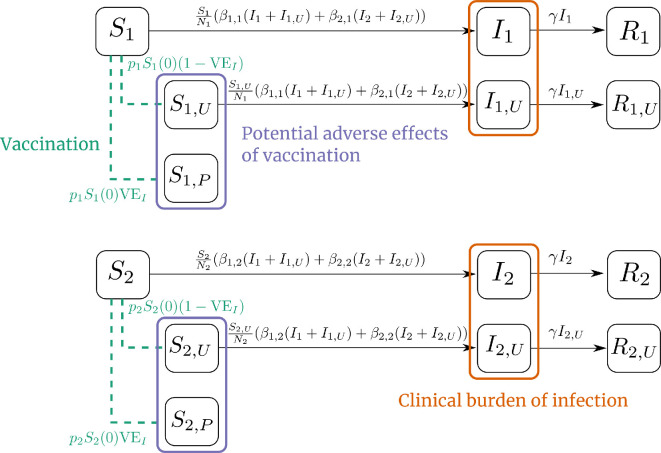
Compartmental model diagram for the two subpopulations with imperfect ‘all-or-none’ vaccination, indicating which compartments contribute to the components of the loss function. Each group is stratified into susceptible S, infectious I and removed R (i.e. immune due to recovery). Subscripts represent group membership and vaccination status: vaccinated but unprotected U, or vaccinated and protected P. Dashed green lines indicate the proportion initially vaccinated. The purple and orange boxes indicate the compartments contributing to the adverse effects of vaccination and clinical burden.

In our transmission model, unvaccinated individuals are mathematically the same as those who are vaccinated but unprotected (through vaccine failure). Both are equally susceptible to infection and, once infected, equally infectious. That is, we assume no partial protection against infection or onward transmission. We use subscripts to indicate vaccination status. For example, in group i, the members of $S_{i}$ are unvaccinated, the members of $S_{i,U}$ are vaccinated but *unprotected* and the members of $S_{i,P}$ are vaccinated and *protected*. The compartmental transmission model with stratification by group and vaccination status is shown in figure 1. The full system of Ordinary Differential Equations (ODEs) corresponding to the diagram in figure 1 is provided in the electronic supplementary material, equation (S2).

### 2.2. Components of the loss function

To choose the best vaccination strategy (choice of $p_{1}$ and $p_{2}$) under a given ethical framework, we measure the *loss* associated with the outcome. To do this, we extend the transmission model described above by adding *clinical burden*. We distinguish between two types of clinical burden: the clinical burden due to severe disease caused by the pathogen (which is reduced by the vaccine-induced protection even when vaccines fail to protect against infection), and the clinical burden due to rare adverse side-effects of vaccination, as described below.

The modelling literature tends to focus on selecting interventions that minimize the aggregate clinical burden. However, there is a growing recognition that this should also consider the equity of clinical burden across the population. As such, we consider two measures of equity across the two populations: equity of burden from infection outcomes, and equity of burden from the adverse impacts of vaccination. Below, we describe a family of loss functions. Each loss function encodes an *ethical framework*, which we define for the purposes of this study as a precise prioritization of the values we include (see below for details).

#### 2.2.1. Loss function components: clinical burden

The clinical burden due to infection in group i (e.g. the total number of days members of group i are hospitalized) is $L_{i}^{I}$. This accounts for the infections among both unvaccinated individuals and those who are vaccinated but unprotected:


(2.1) $$\begin{array}{lcr} & L_{i}^{I}=C_{i}^{I}[I_{i}(t_{f})+R_{i}(t_{f})]+C_{i,U}^{I}[I_{i,U}(t_{f})+R_{i,U}(t_{f})]\;, & \end{array}$$

where $I_{i}(t_{f})+R_{i}(t_{f})$ and $I_{i,U}(t_{f})+R_{i,U}(t_{f})$ are the numbers of unvaccinated and vaccinated–unprotected individuals infected in group i over the time horizon $t_{f}$, and $C_{i}^{I}$ and $C_{i,U}^{I}$ are the average clinical burden for unvaccinated and vaccinated–unprotected members of group i. We allow for the vaccine to reduce the severity of breakthrough infections by specifying $C_{i,U}^{I}=C_{i}^{I}(1-{\text{VE}}_{SD|I})$. The term ${\text{VE}}_{SD|I}$ is a scaling factor for the vaccine’s efficacy against severe disease, given infection. The aggregate clinical burden due to infection in both groups is


(2.2) $$\begin{array}{lcr} & L^{I}=L_{1}^{I}+L_{2}^{I}\;. & \end{array}$$

To include the clinical burden of adverse effects of the vaccine in our decision framework, we compute the expected clinical burden due to vaccination in group i as


(2.3) $$\begin{array}{lcr} & L_{i}^{V}=C_{i}^{V}V_{i}\;, & \end{array}$$

where $C_{i}^{V}$ is the average clinical burden (e.g. expected days in hospital) due to adverse effects of vaccine for a member of group i, and $V_{i}=N_{i}p_{i}$ is the total number of vaccinations administered to members of group i. The net clinical burden associated with vaccination is


(2.4) $$\begin{array}{lcr} & L^{V}=L_{1}^{V}+L_{2}^{V}\;. & \end{array}$$

Combining the contributions of infection-induced and intervention-induced clinical burden, we get the aggregate clinical burden $L_{CB}$, which is given by


(2.5) $$\begin{array}{lcr} & L_{\text{CB}}=L^{I}+L^{V}\;. & \end{array}$$

We note that this aggregate clinical burden is a typical target of optimization when selecting vaccination strategies. To account for considerations of equity, we need to consider another component to the loss function as described below.

#### 2.2.2. Loss function components: equity

Here, we consider an equitable vaccination strategy to be one in which the *per capita* clinical burden is the same for both groups. This is in contrast to prioritizing equity of vaccine distribution, which would see the vaccine supply spread uniformly across all members of the population.

We start by considering what outcome would represent equality of *per capita* burden across groups. If the population shared clinical burden due to infection uniformly, the loss experienced by group i would be $L^{I}N_{i}/N$, since $N_{i}/N$ is the proportion of the population in group i. Similarly, if the burden of adverse effects of vaccination were uniformly distributed, the loss experienced by group i would be $L^{V}(N_{i}/N)$.

To quantify how equitable an intervention is, we measure how close the outcome is to equality. We compute the following loss function measuring the equality of infection burden *per capita*


(2.6) $$L_{EI}={\left\|\left(L_{1}^{I}-L^{I}\frac{N_{1}}{N},L_{2}^{I}-L^{I}\frac{N_{2}}{N}\right)\right\|}_{1},$$

where ${\left\|\cdot \right\|}_{1}$ is the $L^{1}$-norm, that is, ${\left\|(x_{1},x_{2})\right\|}_{1}=|x_{1}|+|x_{2}|$. The value of $L_{\text{EI}}$ measures how far the observed distribution of burden $\left(L_{1}^{I},L_{2}^{I}\right)$ is from an equal distribution of burden $\left(L^{I}\frac{N_{1}}{N},L^{I}\frac{N_{2}}{N}\right)$. Similarly, the measure of equality in the distribution of adverse effects of vaccination is given by


(2.7) $$\begin{array}{lcr} & L_{\text{EV}}={\left\|(L_{1}^{V}-L^{V}\frac{N_{1}}{N},L_{2}^{V}-L^{V}\frac{N_{2}}{N})\right\|}_{1}. & \end{array}$$

The terms $L_{\text{EI}}$ (equation (2.6)) and $L_{\text{EV}}$ (equation (2.7)) measure how far away the *per capita* outcomes are from equality for a given combination of $p_{1}$ and $p_{2}$. In the next section, we describe our approach to combining these terms with the aggregate clinical burden, $L_{\text{CB}}$ (equation (2.5)), for a single loss function.

#### 2.2.3. Combined loss function

Note that $L_{\text{CB}}$, $L_{\text{EI}}$ and $L_{\text{EV}}$ take values over different units and ranges. We normalize each of these terms to obtain dimensionless quantities taking values from 0 to 1. Since we are optimizing over a bounded parameter space ($p_{1}$ and $p_{2}$ take values between zero and one), we can numerically compute the range of values for each loss term. This range of values can then be used to normalize the term to take values between zero and one:


(2.8) $$\begin{array}{lcr} & L_{X}^{*}=\frac{L_{X}-\min(L_{X})}{\max(L_{X})-\min(L_{X})}\;, & \end{array}$$

where X represents a loss term (subscript CB, EI and EV), and the asterisk (*) indicates the value has been normalized to the range [0, 1].

Our loss function is a linear combination of the three normalized loss terms with a weighting of the terms specified by $w_{\text{EI}}$ and $w_{\text{EV}}$:


(2.9) $$\begin{array}{lcr} & L=(1-w_{\text{EI}}-w_{\text{EV}})L_{\text{CB}}^{*}+w_{\text{EV}}L_{EV}^{*}+w_{\text{EI}}L_{\text{EI}}^{*}. & \end{array}$$

The value of $w_{\text{EV}}$ corresponds to the priority given to equity in vaccine-induced adverse outcomes, $w_{\text{EI}}$ corresponds to the priority given to equity of infection-induced clinical outcomes and $\left(1-w_{\text{EI}}-w_{\text{EV}}\right)$ is the priority given to aggregate clinical burden due to infection. Note that we require the weights to satisfy the following properties: $0\leq w_{\text{EI}}\leq 1\;,\text{0}\leq w_{\text{EV}}\leq 1$ and $w_{\text{EI}}+w_{\text{EV}}\leq 1$.

**Figure 2.**
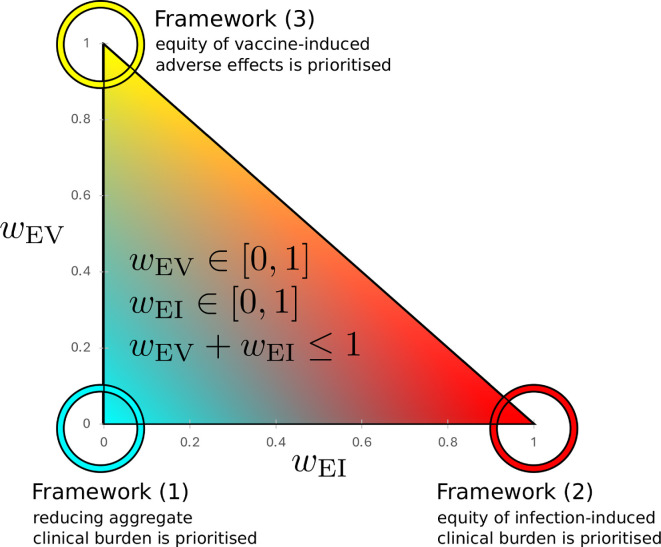
The space of loss functions used to define different ethical frameworks. The two weight coefficients $w_{\text{EI}}$ and $w_{\text{EV}}$ determine the prioritization of three values we consider: (1) aggregate clinical burden, (2) equity of infection-induced clinical burden, and (3) equity of clinical burden from vaccine-induced adverse reactions. The three extreme ethical frameworks we use as examples in our results are indicated by the teal, red and yellow circles at the corners of the triangle.

We can now define an *ethical framework* quantitatively as a choice of $w_{\text{EV}}$ and $w_{\text{EI}}$, which reflects our desired prioritization of these factors. For example, a utilitarian perspective would be realized by small values of $w_{\text{EI}}$ and $w_{\text{EV}}$ (i.e. $1-w_{\text{EI}}-w_{\text{EV}}>w_{\text{EI}}+w_{\text{EV}}$). This would result in the optimization of vaccination primarily minimizing aggregate clinical burden. From the perspective of someone interested in equitable outcomes, one might select larger values for $w_{\text{EI}}$, producing a greater penalty for inequality in the infection-induced burden. Furthermore, a policymaker may have good reason to prioritize equity in terms of harms associated with the intervention (induced harms) over equity in harms associated with infections. Because these harms arise as a directly attributable result of the intervention, they may be more predictable and more easily ascribed to the policy choice than the harms associated with infections. The space of possible ethical frameworks defined by the family of loss functions in equation (2.9) is depicted by the diagram in figure 2.

### 2.3. Example: COVID-19

To investigate the application of our approach under a realistic set of parameters, we compare the results of applying different ethical frameworks to vaccination against COVID-19. Our model is parametrized to match the population of Melbourne, Australia, during the Omicron phase of the pandemic from late 2021 through mid-2022. Motivated by the increased severity of COVID-19 for older adults, including longer average hospital stays [17], we divide our population into groups corresponding to those aged 0−69 years and those aged 70 years or older.

We note that while our parameter estimates are broadly realistic, it is not our intention to exactly reproduce the dynamics of COVID-19 in Australia, which would require a much more detailed model. To understand the behaviour of our model and optimization framework more broadly, we consider two additional scenarios with greater and smaller transmission rates within the population. The lower $R_{0}$ value is closer to Australian estimates of the effective reproductive ratio for Ancestral SARS-CoV-2 [18]. The higher $R_{0}$ value is based on estimates for the Omicron variant if it had been introduced in a completely susceptible population [19].

#### 2.3.1. Parametrization

Parameters used for our model of COVID-19 in Australia during the Omicron period are listed in table 1. These values are derived from census data and parameter estimates from the epidemiological literature, which are summarized in electronic supplementary material, tables S1–S3. The main features of this parametrization are as follows:

— a relatively high reproductive ratio ($R_{0}=3.4$),
— a modest vaccine efficacy against infection,
— a finite time horizon $t_{f}=500\text{ days}$ to capture the policy-relevant time frame,
— a higher (but still modest) vaccine efficacy against severe disease given infection,
— a much higher risk of severe infections in group 2 (representing people 70 years of age or older) than in group 1, and
— a higher risk of adverse impacts of vaccination in group 1 than group 2 (driven by a higher risk of myocarditis in men aged under 30).

**Table 1.** Parameters of the transmission and clinical burden models for COVID-19 based on the estimates from electronic supplementary material, tables S1–S3. See the electronic supplementary material for more details on parameter estimates. *The entries of the contact matrix, $c_{ij}$, are proportional to the number of contacts per day between an individual in group i and members of group j . The remaining constant β is determined by the contact matrix and $R_{0}$ (electronic supplementary material, equation (S1)).

	value	
parameter	group 1	group 2
time horizon, $t_{f}$	500 days	500 days
population size, $N_{i}$	4 395 000	605 000
$R_{0}$	1.5, 3.4, 9	1.5, 3.4, 9
contact matrix*	$c_{11}=0.38N_{1}$	$c_{22}=0.34N_{2}$
	$c_{12}=0.14\;N_{2}$	$c_{21}=0.14\;N_{1}$
γ	0.096	0.096
initially infected, $I_{i}(0)$	1	1
${\text{VE}}_{I}$	0.531	0.531
${\text{VE}}_{SD|I}$	0.627	0.627
expected infection cost, $C_{i}^{I}$	0.00088×2.87	0.0104×7.61
expected vaccination cost, $C_{i}^{V}$	$6\times 10^{-5}\times 5.7$	$2\times 10^{-5}\times 5.7$

#### 2.3.2. Computational method for optimizing the vaccination strategy

To locate optimal vaccination strategies for the model and loss functions described above, we perform a grid search of L over $p_{1}$ and $p_{2}$ with a step size of 0.02.

**Figure 3.**
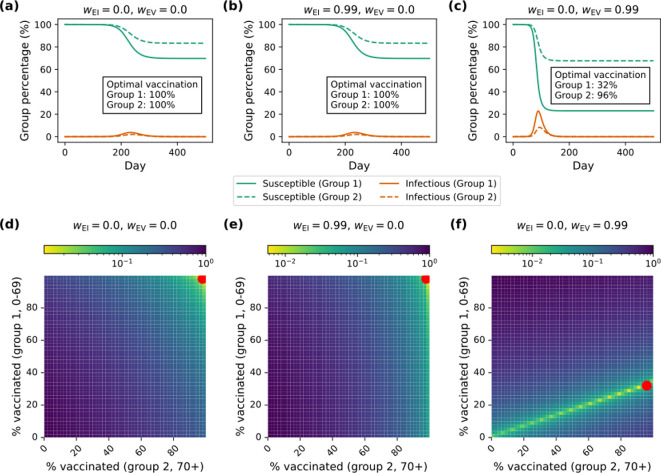
Examples of optimal vaccination strategies ($R_{0}=3.4$). Three different ethical frameworks are shown, one with emphasis on avoiding clinical burden (a,d), one placing priority on equity of infection-induced burden (b,e) and one with emphasis on equity of burden from adverse vaccination impacts (c,f). Trajectories generated under the optimal vaccination strategy for each framework are shown in (a–c), while the corresponding loss surfaces are shown in (d–f). In (d–f), red dots correspond to the optimal vaccination strategy.

Due to discretization effects, the grid search algorithm does not guarantee that the global minimum will be located (this is visible in figure 3f). However, plots of the full loss surface suggest that the true optimum is near enough to the grid search result that our findings are not impacted substantially by discretization effects.

**Figure 4.**
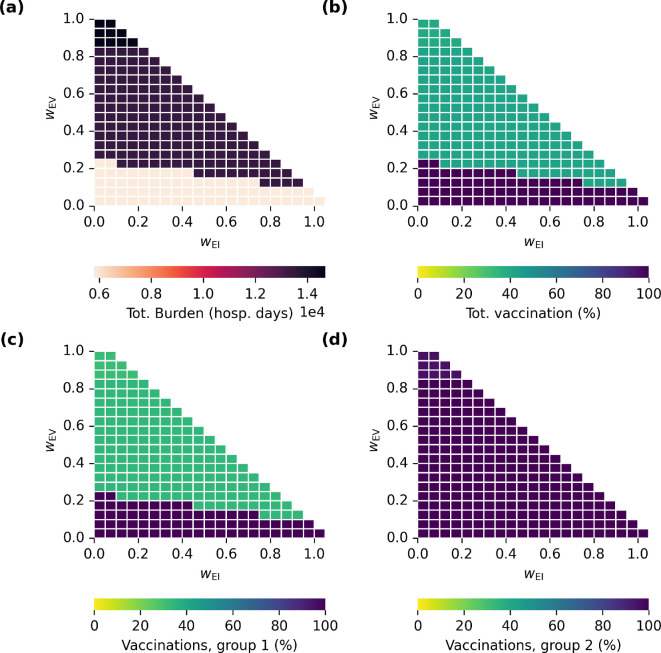
Outcomes of optimal vaccination strategies as functions of the ethical frameworks used to define the loss function. Total clinical burden (days in hospital) is shown in (a). The proportion of the total population vaccinated is shown in (b). The proportion of individuals vaccinated in groups 1 and 2 is shown in (c,d), respectively.

We note that in the case where $w_{\text{EV}}=1$, a unique optimal vaccination strategy does not exist because all equal *per capita* distributions of adverse impacts produce equivalent results (figure 3f illustrates this behaviour). As such, we have excluded the corresponding point from the space of ethical frameworks that we consider below (figure 4).

#### 2.3.3. Ethical frameworks

We consider the optimal vaccination strategy (choice of $p_{1}$ and $p_{2}$) under three specific ethical frameworks, with each representing one of three extremes:

1. Framework 1 ($w_{\text{EI}}=0,w_{\text{EV}}=0$): exclusively prioritizes total clinical burden (figure 3a,d).
2. Framework 2 ($w_{\text{EI}}=0.99,w_{\text{EV}}=0$): prioritizes equity of infection-associated clinical burden almost exclusively, with a small weight fraction 1%, given to aggregate clinical burden (figure 3b,e).
3. Framework 3 ($w_{\text{EI}}=0,w_{\text{EV}}=0.99$): prioritizes equity of clinical burden associated with adverse impacts of vaccination almost exclusively, with a small weight fraction 1%, given to aggregate clinical burden (figure 3c,f).

The positioning of these three frameworks, relative to the overall space of potential loss functions, is shown in figure 2. In frameworks 2 and 3, we set $w_{\text{EI}}$ and $w_{\text{EV}}$ (respectively) close but not equal to one. This ensures the optimization algorithm chooses strategies that produce equitable outcomes while ensuring that if there are multiple equivalently equitable scenarios, the optimization chooses the one corresponding to the lowest clinical burden. We note that these frameworks 2 and 3 are extreme, in the sense that they put little weight on minimizing aggregate clinical burden, but they are useful in illustrating the types of trade-off that may emerge.

## 3. Results

After describing the results of optimizing interventions subject to loss functions corresponding to frameworks 1−3, we examine intermediate weightings over a broad range of values for $w_{\text{EI}}$ and $w_{\text{EV}}$. This allows us to characterize the decision space (figure 2) in terms of the chosen vaccination strategies and the associated outcomes. By doing so, we identify potential trade-offs between equity and aggregate clinical burden. Below, we provide this analysis for three scenarios, $R_{0}=3.4$ representing the COVID-19 Omicron variant and two additional scenarios with $R_{0}=1.5$ and $R_{0}=9$.

### 3.1. COVID-19 example ($R_{0}=3.4$)

Epidemic trajectories under the optimal allocation of vaccines with $R_{0}$ = 3.4 are shown in figure 3a–c for frameworks 1, 2 and 3, respectively. To gain further insight into why these vaccination strategies are optimal under each of the ethical frameworks, figure 3d–f shows the global loss function equation (2.9) as a function of the vaccination proportions. The optimal proportions (indicated with a red dot in figure 3d–f) are the vaccination strategy used in the corresponding trajectory shown in figure 3a–c, respectively.

Full vaccination (100% in groups 1 and 2) is selected as the optimal strategy for frameworks 1 and 2. The trajectories in figure 3a,b demonstrate that, due to our parametrization of vaccine efficacy and pathogen transmissibility, herd immunity is not achievable (substantial epidemics occur, even with 100% vaccination).

In contrast, because group 1 is at higher risk of adverse side-effects from vaccination, optimization under framework 3 leads to vaccinating fewer individuals in group 1 and more infections in both groups 1 and 2 (figure 3c,f). We note that the linear trend in figure 3f (with a local minimum at $p_{1}\approx 0.32$) follows the ratio established by the clinical burden due to adverse reactions: $C_{1}^{V}/C_{2}^{V}=1/3$. This indicates a trade-off between equity in the adverse impacts of vaccination and aggregate clinical burden.

Next, we examine how the optimal vaccination strategy differs over a broader range of value prioritization. The results of optimal interventions over intermediate values of $w_{\text{EI}}$ and $w_{\text{EV}}$ are shown in figure 4. The heatmap in figure 4a shows the total clinical burden obtained when using the optimal vaccination for those values of $w_{\text{EI}}$ and $w_{\text{EV}}$. The corresponding proportion of the total population vaccinated is shown in figure 4b. The allocation of vaccines is shown in figure 4c,d for group 1 and group 2, respectively.

Examining how the optimal vaccination strategy changes as a function of $w_{\text{EI}}$ and $w_{\text{EV}}$ allows us to understand how sensitive our allocation decision is to different prioritization of values. Over almost all of the parameter space in $w_{\text{EI}}$ and $w_{\text{EV}}$, there are only two vaccine allocation strategies that are identified as optimal. These are shown in figure 4c,d, which provide optimal choice of vaccine allocation strategy for groups 1 and 2, respectively, as functions of $w_{\text{EI}}$ and $w_{\text{EV}}$.

Given our chosen parametrization based on SARS-CoV-2 and COVID-19, the results shown in figure 4c,d suggest the optimal vaccination strategy is insensitive to the priority given to equity in infection burden ($w_{\text{EI}}$). However, there is a clear decision boundary in figure 4c as the weight put on equity in vaccine adverse effects ($w_{\text{EV}}$) increases. At low levels, the optimal strategy is to vaccinate everyone in both groups 1 and 2; however, as more weight is placed on $L_{\text{EV}}$, there is a transition to vaccinate approximately 30% of group 1, leading to a higher clinical burden (figure 4a).

### 3.2. Greater and smaller $R_{0}$

**Figure 5.**
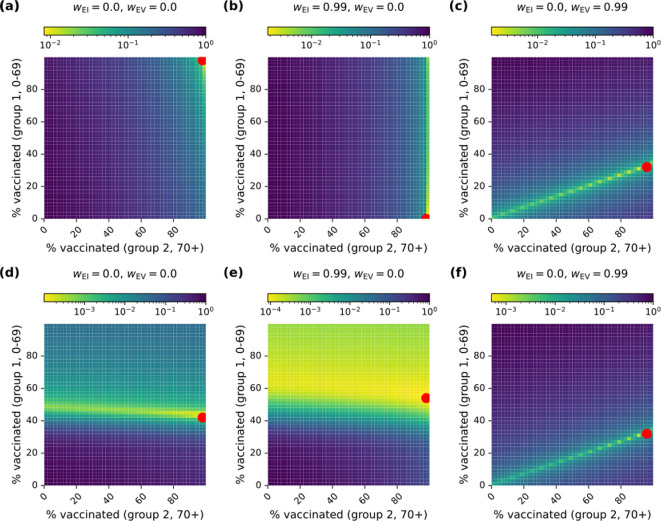
Examples of optimal vaccination strategies with greater and smaller values of $R_{0}$. (a–c) The loss surface for $R_{0}=9$, and the red dots correspond to the optimal vaccination strategy for each ethical framework. (d–f) The loss surface for $R_{0}=1.5$.

Here we examine scenarios in which the reproductive ratio is greater ($R_{0}=9.0$) or smaller ($R_{0}=1.5$) than the value $R_{0}=3.4$ used in §3.1. Greater $R_{0}$ means that the epidemic now spreads more readily, and substantial indirect protection is not achievable. On the other hand, for smaller $R_{0}$, it is now possible to avert an epidemic if sufficiently large numbers of people are vaccinated. The trajectories corresponding to the optimal vaccination strategies under frameworks 1−3 are shown in electronic supplementary material, figures S1 and S3, for high and low $R_{0}$, respectively. Corresponding loss surfaces as functions of vaccination strategy are shown in figure 5.

With high transmissibility ($R_{0}=9$), a trade-off appears between equity of infection-associated clinical burden and aggregate clinical burden (figure 5a,b). While frameworks 1 and 2 both recommend vaccinating all individuals in group 2, the optimal strategies for group 1 are different: framework 1 recommends vaccinating all individuals in groups 1 and 2 (figure 5a). Alternatively, framework 2 recommends no vaccinations for group 1, withholding available resources (figure 5b). Framework 3 does not change with higher $R_{0}$ because it only accounts for clinical burden induced by the vaccine (figure 5c).

For a lower reproductive ratio ($R_{0}=1.5$), a different type of trade-off between aggregate burden and equity of infection-induced burdens is observed (figure 5d,e). Because it is possible to prevent widespread transmission due to the lower reproductive ratio, framework 1 selects a strategy in which large epidemics are prevented. Vaccines are allocated such that everyone in group 2 is vaccinated and enough people in group 1 are vaccinated to prevent large epidemics. On the other hand, while framework 2 still vaccinates everyone in group 2, it gives additional vaccines to group 1, beyond what is needed to prevent large epidemics. This provides additional indirect protection to those in group 2 for whom the vaccine failed, at the cost of vaccine-induced clinical burden in group 1. Here, group 1 experiences additional harm, so that members of group 2 can be protected from infection.

**Figure 6.**
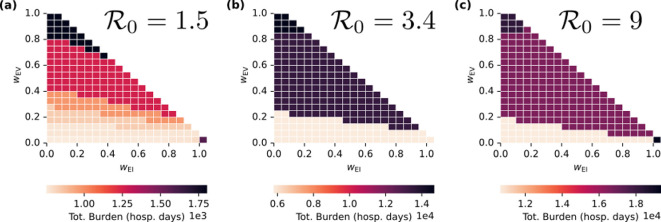
Total clinical burden (days in hospital) from optimal vaccination strategies as functions of the ethical frameworks used to define the loss function for $R_{0}=1.5$ (a), $R_{0}=3.4$ (b) and $R_{0}=9$ (c).

A comparison of aggregate clinical burden associated with optimal strategies over a broader range of ethical frameworks for each value of $R_{0}$ we examined is shown in figure 6. More complete sets of outcome results for low- and high-$R_{0}$ scenarios in the format of figure 4 are shown in electronic supplementary material, figures S2 and S4.

For low and high $R_{0}$, trade-offs between equity of infection-associated burden and aggregate burden are confined to extreme values of values of $w_{\text{EI}}$ (bottom right corners of the triangles in figure 6a,c). For the case of high $R_{0}$, we note that this trade-off could be considered a violation of the principle of non-maleficence, in that the choice is made to withhold available resources in order to promote equity of outcomes between two groups (by allowing the more well-off group to be subject to preventable burdens). In the case of low $R_{0}$, the choice is made to vaccinate group 1 more than is necessary to prevent infections, in order to protect group 2 and achieve higher equity of infection-associated burden. This choice produces net harm in group 1 and could, therefore, also be considered a violation of non-maleficence. However, this trade-off is more complex than the one observed for high $R_{0}$ because benefits are provided to group 2 as a result of harms inflicted on group 1.

For values of $w_{\text{EI}}\leq 0.95$ and $w_{\text{EV}}=0$, no trade-off exists between aggregate clinical burden and equity of infection burden (there are no decision boundaries horizontally across the bottom line of the plots in figure 6). On the other hand, clear decision boundaries exist as a function of $w_{\text{EV}}$ for all three values of $R_{0}$. As $w_{\text{EV}}$ increases past 0.1, a higher aggregate burden is observed because fewer of the individuals in group 1 are vaccinated (figure 4c and electronic supplementary material, figures S4c and S2c). Here, the vaccines have been distributed such that the burden of adverse events is distributed equitably between the two groups, despite the fact that this decision leaves many individuals without immunization. This scenario is analogous to prioritizing the equitable distribution of resources (this situation arises precisely if the adverse event rate *per capita* is identical in the two groups, $C_{1}^{V}=C_{2}^{V}$). When two groups have different risk profiles, equitable distribution of vaccines can lead to inequitable outcomes.

## 4. Discussion

We have presented an approach to defining loss functions for models of infectious disease interventions that include ethical values related to public health. These loss functions correspond to ethical frameworks, which, in the context of our study, we define as prioritizations of ethical values, including reduction of aggregate clinical burden and promotion of equity in clinical burdens between groups. By examining the strategies selected under different frameworks, we investigate potential value trade-offs. We constructed a simple model of infectious disease transmission and intervention (in this case, vaccination). We then defined a loss function to measure the aggregate clinical burden and inequity resulting from the intervention. We used the loss function to systematically locate the optimal intervention (i.e. vaccination strategy) for different prioritizations of values.

There is a growing awareness that selecting interventions based on projections of aggregate clinical burden can produce inequitable outcomes or exacerbate existing inequities (e.g. [20]). In our example based on COVID-19, we found for ethical frameworks that put a substantial weight on minimizing aggregate burden, the optimal strategy was to vaccinate everyone, starting with those at most risk of severe disease. This was the optimal strategy even when some weight was given to either of the equity measures we considered. We only found a trade-off between minimizing aggregate clinical burden and equity of infection burden when the ethical framework placed negligible weight on aggregate burden. For $R_{0}=9$, protective vaccination was withheld from one group in the interests of equity, while for $R_{0}=1.5$, extra vaccines were given to one group who did not need them (producing adverse reactions) in order to protect the other group from infection. For the COVID-19 example with $R_{0}=3.4$, there was no trade-off even in the extreme case of framework 2 (putting almost all priority on equity of infection burden). This occurred because indirect protection of group 2 was achieved by providing vaccines to group 1, and the disease was sufficiently infectious that these vaccines benefited group 1 as well.

Our findings agree qualitatively with those of Stafford *et al.*, who found (in a model of COVID-19 in the United States) that aggregate mortality and outcome equity could be jointly optimized as long as total vaccine coverage exceeded 30% [21]. While this outcome may be robust for scenarios describing vaccination against COVID-19, we expect it to be context-specific. For example, a robust trade-off between equity of clinical burden and total clinical burden might be expected in scenarios where transmission is driven by subpopulations that are not at high risk of severe disease. In such cases, preventing low-risk infections may produce a high overall reduction in case numbers (through indirect protection), but could lead to an inequitable distribution of burdens because the group with high outcome risk is not directly protected. Such an alternative outcome would be consistent with the findings of Goldstein *et al.*, who investigated vaccine allocation strategies using a model of H1N1 influenza in the United States. They found that optimal strategies supported vaccination of children, who are at low risk of adverse outcomes but are disproportionately contagious if infected [22].

We consistently observed a potential trade-off between aggregate clinical burden and the equitable distribution of intervention-induced harms, with aggregate clinical burden increasing as $w_{\text{EV}}$ increased (figure 6). This can be considered a generalization of the well-known trade-off between aggregate benefit and the uniformity of resource allocation in populations with heterogeneous risk profiles [3].

To put these observations about trade-offs between equity and aggregate burden into context, we note that frameworks 2 and 3 are not intended to reflect the intentions of those who advocate for equity in public health (where it is only one of many values that are jointly considered). Neither should the analysis and discussion of our results be interpreted as advocacy in favour of any given strategy of decision-making. Our equity calculations (equations (2.6) and (2.7)) quantify only the equality of *per capita* outcomes between groups. Therefore, our quantification of equity does not incorporate nuanced definitions from the ethics literature that address the ‘levelling down’ critique of egalitariansim [23,24]. As specified, frameworks 2 and 3 are capable of accepting scenarios in which everyone is worse off than they could be, given the resources available. As long as the relevant *per capita* outcomes are as similar across population groups as possible, these frameworks are satisfied. More formally, when defining equity, we ignore any explicit concept of justice in the sense used by Wolff [23] or Christiano & Braynen [24], where justice translates to the requirement that maximization of equity occurs only among a set of attainable options that are Pareto-equivalent in terms of aggregate benefit.

Another key observation was of abrupt decision boundaries in the space of value prioritization (observable in figures 4 and 6b,c). For scenarios with $R_{0}=3.4$ and $R_{0}=9$, optimal strategies tended to correspond to one of the three extreme ethical frameworks: frameworks 1−3. This abruptness in the decision landscape is not guaranteed and depends on the gradients of the loss surfaces for each value considered. For example, with $R_{0}=1.5$, the decision landscape was more gradual, with a smoother transition of outcomes between strategies (figure 6a and electronic supplementary material, figure S4). One interpretation of this feature is that, for the scenarios with $R_{0}=3.4$ and $R_{0}=9$, ethical frameworks with nuanced balances of different values are not easily distinguished from extreme frameworks that mainly prioritize individual values. On the other hand, the smoother gradient of outcomes with $w_{\text{EI}}$ and $w_{\text{EV}}$ for $R_{0}=1.5$ indicates that ethical frameworks with subtly different value priorities would produce distinct outcomes.

The simplicity of our transmission model implementation introduces several limitations. For example, a fixed parametrization of a deterministic model cannot address problems of uncertainty in model parameters or stochastic effects in the transmission process. Further, our assumption that vaccines are distributed prior to the initial outbreak means we could not investigate how the logistics of distribution may change the outcomes of a given strategy.

Our assessment of equity was also limited because we elected to stratify the population into only two groups (those under the age of 70 and those 70 or older). Age was chosen because it is widely recognized as clinically relevant to our example of COVID-19, but other important characteristics could be used as well, such as socio-economic status or ethnicity [21]. The choice of which population strata to include and which outcomes to evaluate should reflect the ethical priorities of interest to decision makers. For example, if economic impact and pre-existing economic disadvantage are major priorities alongside clinical burden, then projections of outcomes associated with lost income may be needed as well (e.g. [25]).

While quantifying equity over more complex population strata and multiple outcomes is a technical challenge, recent methods have been developed to achieve this through the adaptation of Gini coefficients to stratified epidemic outcomes [26]. Individual-based welfare functions, as implemented by Adler *et al.* in prioritarian social welfare analysis, provide another approach to flexible assessment of equity considerations along multiple dimensions [13,25]. However, unlike prioritarian social welfare analysis, our approach emphasizes the capacity to encode the separate value components of ethical frameworks into the final loss function. This separability was essential to our analysis of the individual value components, and we found it useful in terms of understanding the limitations and implications of frameworks built around single value considerations (i.e. our ‘extreme’ frameworks 1−3).

While our study is constrained by our choice of model, the approach is model-agnostic. Any system that can quantify the outcomes of prospective policy decisions could be compatible with loss functions like those we demonstrate here. The principal contribution of this study is to establish a link between frameworks of public health ethics and the types of models used to inform intervention policy.

We anticipate future methodological challenges when incorporating more comprehensive systems of values into increasingly complex models. To establish a proof of principle, we have treated both of these aspects with relative simplicity, which results in a number of limitations. These are the other value considerations that were not included in our loss function. For example, inequity in access to resources; burdens arising from social interventions, such as stay-at-home orders; and unintended effects such as delayed medical treatments [27]. The inclusion of state-of-the-art multi-criteria decision-making approaches (e.g. [28]) would allow a more realistic specification of values and constraints and an enhanced capacity to capture nuanced aspects of ethical frameworks. We note that the emphasis on evaluation of *outcomes* is implicit in the construction of our loss functions, which restricts our methods to values that can be included in consequentialist ethical frameworks (those which concern the outcomes of interventions, rather than the process which produced those outcomes). There are many ways in which the use and development of models could be relevant to procedural values in public health (e.g. transparency, accountability and trust), but those are outside the scope of this proof-of-concept.

While many ethical values in public health may potentially be included within outcome-based approaches such as this, some may be out of scope because the relevant types of outcomes are not feasible to quantify. For example, our loss function ignores the preferences of the individuals in the population; it only considers what level of vaccination would be optimal based on quantifiable clinical burdens. Relatedly, it cannot include any consideration of whether or not coercion would be justified to achieve intervention targets.

## 5. Conclusion

Frameworks of public health and biomedical ethics often seek to associate weights with principles through the process of value balancing [29]. Our approach quantifies the effects of such weights in the context of a decision process made explicit by a mathematical model and optimization problem. This facilitates exploration over the space of alternative weightings, to identify the presence, absence and magnitudes of potential value trade-offs. While it is beyond the scope of our method to offer specific guidance for selecting the value weights, our study provides insight into how robust the decision landscape is to changes in these weights. This work provides an approach to understanding how sensitive an optimal intervention strategy may be to a chosen set of value weights. By demonstrating value balancing in the context of a model with policy-relevant features, we hope to stimulate further work linking ethical frameworks to policy-relevant models of infectious disease interventions.

## Data Availability

All code and data associated with this paper can be found on the Zenodo repository [30]. Electronic supplementary material is available online [31].
